# Early onset monocular hydroxychloroquine maculopathy in a systemic lupus erythematosus patient with history of central retinal artery occlusion: a case report

**DOI:** 10.1186/s12886-022-02657-8

**Published:** 2022-11-14

**Authors:** Ahmed Ameen Ismail, Sherin Hassan Sadek, Ragai Magdy Hatata

**Affiliations:** Department of Ophthalmology, Faculty of Medicine, Fayoum University, Al-Tadreeb Square, Al Fayoum, Egypt

**Keywords:** Hydroxychloroquine maculopathy, HCQ, SLE, CRAO, Bull's eye, Case report

## Abstract

**Background:**

Hydroxychloroquine is a widely used medication for various clinical conditions mainly rheumatological and dermatological autoimmune diseases e.g. systemic lupus erythematosus, rheumatoid arthritis and psoriasis. While it is considered a safe medication, it is well-established that it can cause retinal toxicity i.e. HCQ maculopathy. Guidelines for HCQ retinal toxicity screening include factors like body weight, daily dose, duration, systemic diseases and retinal diseases. In this case study, we report a specific association between CRAO as a retinal disease and early onset HCQ maculopathy in a SLE patient.

**Case presentation:**

A 42-year-old Caucasian female SLE patient presented with a complaint of gradual progressive painless diminution of vision in the left eye that started 16 months earlier. Clinical evaluation of the patient revealed a history of sudden profound painless diminution of vision in the same eye 18 months earlier after which the patient experienced only partial improvement of vision. That episode of sudden diminution of vision was attributed to left CRAO, complicating SLE-related thrombophilia, confirmed by fundus fluorescein angiography. Based on that diagnosis, the patient had been prescribed HCQ. At the time of presentation, fundus examination revealed left bull's eye maculopathy and right normal fundus. Therefore, a diagnosis of HCQ maculopathy in the left eye was made after exclusion of other causes of unilateral bull's eye maculopathy.

**Conclusion:**

Our case study is the first to report an association between CRAO as a specific retinal disease and early onset of HCQ maculopathy in a SLE patient. The unilateral bull's eye presentation which occurred in the eye with CRAO after only 16 months of HCQ treatment highly suggests that CRAO is probably the cause of such unusually early maculopathy. This case report highlights the importance of retinal diseases as risk factors for HCQ maculopathy. It also points out the lack of specific evidence concerning the association between specific retinal diseases and HCQ maculopathy.

## Background

HCQ is originally an antimalarial drug but was quickly noted for its anti-inflammatory and immunomodulatory effects and became a cornerstone drug in the armamentarium of rheumatologists in diseases like SLE and rheumatoid arthritis. However, it has some adverse events including maculopathy [1]. The incidence of HCQ maculopathy was the subject of various retrospective studies and is generally estimated to be < 1% at 5 years, < 2% at 10 years and as high as 20% at 20 years [1–5]. The higher reported incidence in more recent studies maybe due to more sensitive screening tools which include spectral domain optical coherence tomography (SD-OCT), visual field (VF), multifocal electroretinography (mfERG), fundus autofluorescence (FAF) [4]. Among these screening tools, OCT and FAF detect structural changes which tend to occur later in the course of toxicity and are usually irreversible [1]. While VF and mfERG are considered more sensitive and detect much earlier functional changes that precede irreversible structural damage [6]. Various mechanisms have been proposed for HCQ maculopathy, the most notable of which is the accumulation of HCQ in the melanin-laden RPE leading to RPE dysfunction in the course of years with the end result of RPE and subsequent photoreceptor loss [7]. These advanced RPE changes are visible clinically through fundus examination culminating in its advanced forms as bull's eye maculopathy [8]. As we mentioned earlier, the incidence of HCQ maculopathy is generally low, but the fact that not all patients develop maculopathy despite similar cumulative weight-adjusted drug dose raised the interest in determining risk factors for HCQ maculopathy. Most important factors include: body weight especially real body weight, daily dose, duration, systemic diseases mainly hepatic and renal impairment and retinal diseases. The latest AAO guidelines concerning the screening for HCQ maculopathy decreased the safe dose from 6.5 mg/Kg/day to 5 mg/Kg/day based on real body weight. The AAO also recommended baseline ophthalmological assessment prior to commencing HCQ then yearly from the fifth year onwards. The guidelines recommend more frequent screening visits for patients at higher risk or who start to develop signs of toxicity. The AAO guidelines stated retinal diseases as a risk factor for HCQ maculopathy acknowledging nonetheless that no specific evidence exists to support this recommendation [9]. This case study is, as far as we know, the first to report an association between CRAO and early onset HCQ maculopathy. This may warrant closer screening of patients with retinal vascular occlusions in the course of HCQ treatment. Furthermore, retinal vascular occlusive events have a much higher incidence in various diseases that are frequently prescribed HCQ e.g. SLE which makes vigilance for retinal vascular occlusions in the context of HCQ treatment even more important [10].

## Case presentation

**Fig. 1:**
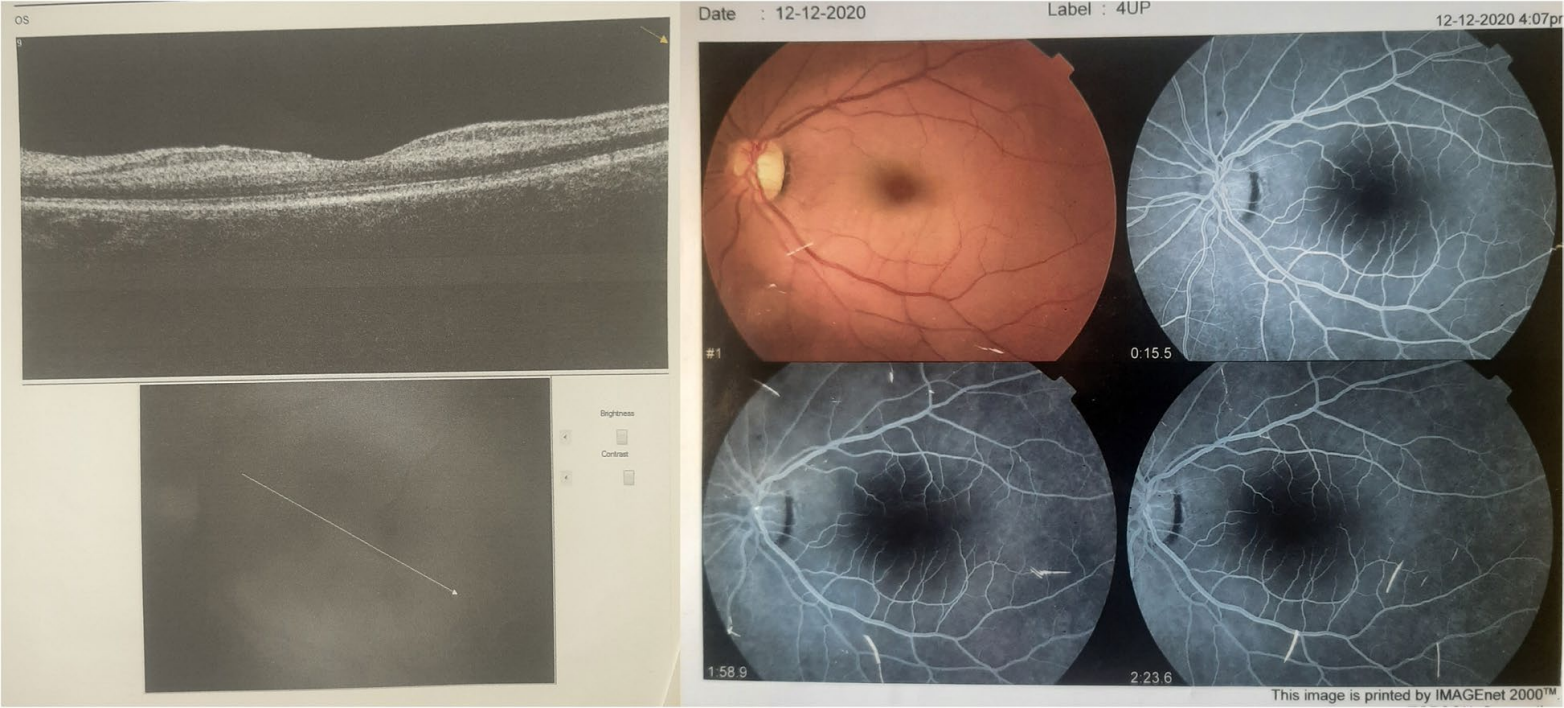
SD-OCT macula of the left eye (left), Fundus photography and FFA of the left eye(right) one week after the acute drop of vision in the left eye that had occurred 18 months prior to presentation to our clinic. OCT shows diffuse, intense hyperreflectivity of the inner retinal layers. Fundus photograph and FFA show the cherry red spot appearance and the surrounding whitening of the macular area and the corresponding widening of FAZ (right)

**Fig. 2:**
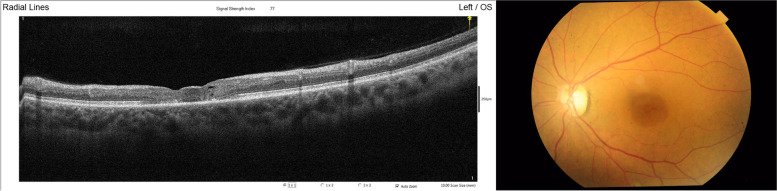
Fundus photography of the left eye (right) and SD-OCT macula of the left eye (left) at the time of presentation to our clinic 16 months after HCQ treatment. Fundus photograph shows the bull's eye appearance in the macula with a darkly pigmented parafoveal ring. OCT shows the foveal and parafoveal outer retinal defect affecting RPE, ELM, IS/OS. There are also intraretinal degenerative cyst, irregular foveal contour and generalized thinning of neurosensory retina

**Fig. 3:**
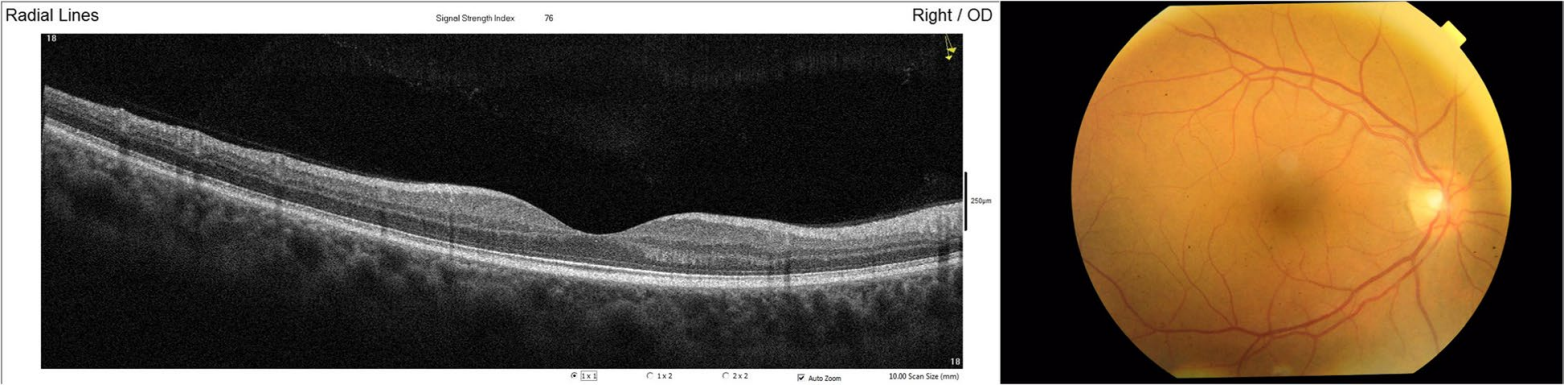
Normal SD-OCT macula of the right eye (left) and normal fundus photography of the right eye (right) at time of presentation to our clinic 16 months after HCQ treatment

**Fig. 4:**
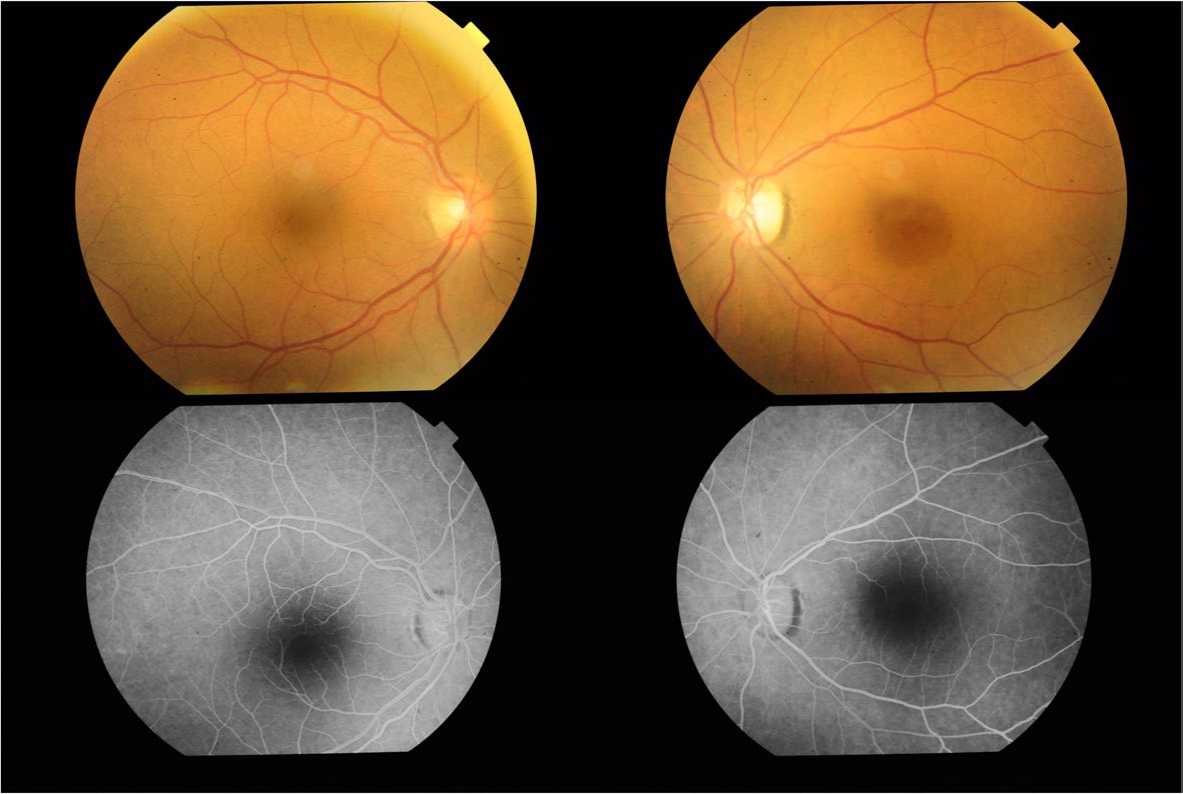
Bilateral fundus photography and FFA at the time of presentation to our clinic 16 months after HCQ treatment. Fundus photography shows the bull's eye appearance in the macula of the left eye, the attenuated arteriolar caliber and exaggerated choroidal pattern in the left eye compared to the right eye due to generalized atrophy of the inner retina secondary to the old CRAO. FFA shows right normal angiogram and left FAZ widening and two concentric parafoveal rings, an inner subtle hyperfluorescent ring representing RPE window defect and an outer larger hypofluorescent ring corresponding to the darkly pigmented parafoveal ring seen in the fundus photograph

A 42-year-old female patient presented in June 2022 with a complaint of gradual progressive painless diminution of vision in the left eye that started 16 months earlier. Clinical history revealed an episode of sudden, profound, painless diminution of vision in the same eye that had occurred 18 months earlier in December 2020. At the time of that episode, visual acuity in the left eye was as low as hand motion, but gradually improved over a period of 4 months to reach 3/60. However, after that initial partial improvement, visual acuity in the left eye progressively deteriorated from 3/60 to become 1/60 at the time of presentation. Through medical history and earlier investigations i.e. FFA, SD-OCT macula (Fig. 1), we concluded that the episode of acute drop of vision in the left eye was caused by left CRAO for which the patient had undergone an extensive systemic workup that established a diagnosis of SLE according to EULAR/ARC criteria with positive ANA, positive anti-Cardiolipin Ab, positive beta2 Glycoprotein Ab, fever, thrombocytopenia and non-cicatricial alopecia [11]. The rheumatologist prescribed the patient HCQ at a dose of 400 mg/day which is equivalent to 5.3 mg/kg/day (slightly above the recommended daily dose of 5 mg/kg/day by the AAO revised guidelines 2016) and oral steroids with varying doses throughout the course of treatment ranging between 20 and 60 mg/day. At the time of presentation, ophthalmological examination revealed BCVA 6/6 OD and 1/60 OS, unremarkable anterior segment examination, right normal fundus, left bull's eye maculopathy and temporal optic disc pallor with attenuated retinal vessels (Fig. 2).SD-OCT macula was performed which revealed foveal and parafoveal outer retinal defects involving the RPE, ELM, IS/OS as well as intraretinal degenerative cysts and generalized thinning and atrophy of the neurosensory retina in the left eye (Fig. 2) while the right eye was normal (Figs. 3 and 4). Therefore, a diagnosis of left advanced HCQ maculopathy was made after exclusion of other possible causes of unilateral bull's eye maculopathy e.g. resolved unilateral acute idiopathic maculopathy (UAIM), subretinal fibrosis and uveitis syndrome (SFU), asymmetric cone or cone/rod dystrophy, benign concentric annular macular dystrophy, Stargardt's disease and traumatic maculopathy. These causes were excluded because none of them matches the clinical picture of our case for variable reasons e.g. the negative family history and the strictly unilateral involvement with completely normal right eye with BCVA 6/6 and intact color vision, excludes dystrophies. Moreover, the insidious rather than acute onset, the absence of signs of uveitis, the strict monofocal foveal and parafoveal involvement as opposed to multifocality, the regular oval edges of bull's eye rather than irregular pigmented edges, exclude UAIM and SFU as possible etiologies. Furthermore, the negative history of ocular or head trauma excludes traumatic maculopathy. Consequently, a recommendation to stop HCQ was conveyed to the rheumatologist who shifted the patient to Leflunomide.What is atypical about this case is the rather early onset maculopathy which manifested in the advanced form of bull's eye maculopathy after only 16 months of HCQ treatment compared to the reported incidence which is < 1% at 5 years. Another unusual aspect is the strictly unilateral involvement where the left eye shows advanced maculopathy while the right eye is normal. We assume that the early onset monocular HCQ maculopathy may be related to the CRAO that affected the same eye prior to HCQ treatment.

## Discussion and conclusion

HCQ has become a mainstay in the management of myriad autoimmune diseases. It proved effective in decreasing morbidity and mortality in various autoimmune conditions e.g. SLE with a relatively safer profile compared to other options like long term systemic steroids and immunosuppressants [7, 12]. However, it doesn't come without adverse effects. One of the well-established complications of HCQ treatment is retinal toxicity namely HCQ maculopathy [2]. With the increasing awareness of the condition together with the advent of more sensitive diagnostic tools e.g. mfERG, VF, FAF, SD-OCT; we are able to detect HCQ maculopathy much earlier which is crucial for the long term visual prognosis since late HCQ-induced damage tends to be irreversible and sometimes even progressive despite drug discontinuation [1]. Multiple retrospective studies investigated the incidence of HCQ maculopathy with varying results but it is generally reported to be < 1% at 5 years, < 2% at 10 years, < 20% at 20 years highlighting the duration of treatment as an important risk factor [4]. These studies associated maculopathy to body weight and adjusted body weight taking into account the pharmacokinetic properties of HCQ [9]. Also the daily dose and duration were among the important factors since they are direct contributors to drug accumulation which has been hypothesized to be at the core of HCQ maculopathy pathogenesis where HCQ accumulation in the melanin-laden RPE causes RPE dysfunction and trophic changes with consequent photoreceptor loss [7]. Other factors that might contribute to a higher incidence of HCQ maculopathy include systemic diseases e.g. renal and hepatic impairment and retinal diseases. Based on results from retrospective studies, the AAO formulated guidelines for HCQ retinal toxicity screening where it accounted for the various reported risk factors. The latest recommended daily dose according to the AAO is 5 mg/Kg/day which adjusts for factors of body weight and daily dose. The guidelines also recommended baseline ophthalmological assessment prior to commencing HCQ and yearly thereafter starting from the fifth year of treatment which acknowledges the cumulative nature of HCQ maculopathy pathogenesis as well as the reported incidence which is as low as 1% at 5 years [4, 9]. However, more frequent follow-ups are required for patients with systemic diseases e.g. renal or hepatic impairment as well as retinal diseases. When it comes to retinal diseases as a risk factor for HCQ maculopathy, the AAO 2016 revised guidelines state that there is no specific evidence to confirm such a risk and that such a recommendation concerning retinal diseases is based on the rationale of not adding a retinotoxic medication to an already compromised retina [9]. The lack of specific evidence concerning retinal diseases as risk factors for HCQ maculopathy thus constitutes a gap in our knowledge and therefore deprives our clinical practice concerning HCQ and retinal diseases from evidence-based robustness. This case study addresses this lack of specific evidence by reporting a direct association between a specific retinal disease namely CRAO and early onset HCQ maculopathy where bull's eye maculopathy developed monocularly after only 16 months of treatment in the eye with CRAO. Various cases of HCQ maculopathy in SLE patients have been reported but they were all bilateral and occurred after at least 6 years of HCQ treatment [13–15].We assume that the early onset unilateral HCQ maculopathy may be related to the central retinal artery occlusion that affected the same eye prior to commencing HCQ. We theorize that the early development of outer retinal defects with RPE and photoreceptor loss may be due to a decrease in Muller-cell derived neurotrophic signals to photoreceptors secondary to Muller cell loss and dysfunction following CRAO. It is reported in literature that neurotrophic factors like brain derived neurotrophic factor(BDNF), ciliary neurotrophic factor(CNTF) and glial cell derived neurotrophic factor(GDNF) decreased the rate of photoreceptor loss in some models of retinal degenerations and phototoxicity [16–19]. Therefore, we hypothesize that the decrease in such glia-dependent neurotrophic factors following CRAO may predispose the retina to HCQ toxicity with rapid loss of photoreceptors leading to the early onset monocular presentation in our case. However, further research is needed to validate such theory. While a single case report is not enough evidence to establish a definitive association, it helps nonetheless guide future large-scale studies towards specific retinal diseases as suspects of increasing risk of HCQ maculopathy. It also conveys a message to clinicians about the need for close and meticulous screening and follow-up of patients with retinal occlusive vasculopathies on HCQ treatment.

## Data Availability

Data is available from the corresponding author on reasonable request.

## Abbreviations

AAO: American Academy of Ophthalmology
Ab: Antibodies
BDNF: Brain Derived Neurotrophic Factor
CNTF: Ciliary Neurotrophic Factor
CRAO: Central Retinal Artery Occlusion
ELM: External Limiting Membrane
EULAR/ACR: European League Against Rheumatism/American College of Rheumatology
FAF: Fundus Autofluorescence
FAZ: Foveal Avascular Zone
FFA: Fundus Fluorescein Angiography
GDNF: Glial Cell Derived Neurotrophic Factor
HCQ: Hydroxychloroquine
IS/OS: Inner Segment/Outer Segment
mfERG: Multifocal Electroretinogram
SD-OCT: Spectral Domain Optical Coherence Tomography
RPE: Retinal Pigment Epithelium
SFU: Subretinal Fibrosis and Uveitis Syndrome
SLE: Systemic Lupus Erythematosus
UAIM: Unilateral Acute Idiopathic Maculopathy
VF: Visual Field
